# Tonic NMDA receptor signalling shapes endosomal organisation in mammalian cells

**DOI:** 10.1038/s41598-020-66071-0

**Published:** 2020-06-09

**Authors:** Oleg O. Glebov

**Affiliations:** 1 Institute of Neuroregeneration and Neurorehabilitation, Qingdao University, Qingdao, 266071 Shandong China; 2 Department of Old Age Psychiatry, Institute of Psychiatry, Psychology & Neuroscience, King’s College London, 16 De Crespigny Park, London, SE5 8AF UK

**Keywords:** Cancer, Membrane trafficking, Endosomes, Ion channels in the nervous system

## Abstract

Calcium signalling through NMDA-type glutamate receptors (NMDARs) plays a key role in synaptic plasticity in the central nervous system (CNS). NMDAR expression has also been detected in other tissues and aberrant glutamate signalling has been linked to cancer; however, the significance of NMDAR function outside of the CNS remains unclear. Here, I show that removal of extracellular calcium rapidly decreases the size of early endosomes in primary human fibroblasts. This effect can be mimicked by blockade of NMDA-type glutamate receptors but not voltage-gated calcium channels (VGCCs), and can also be observed in primary hippocampal neurons and Jurkat T cells. Conversely, in a breast cancer cell line MDA-MB-231 NMDAR blockade results in an increase in endosomal size and decrease in number. These findings reveal that calcium signalling via glutamate receptors controls the structure of the endosomal system and suggest that aberrations in NMDAR-regulated membrane trafficking may be associated with cancer.

## Introduction

Calcium signalling plays a host of important roles in cell function. The overall concentration of Ca^2+^ in the cytosol is generally maintained at an extremely low level, and Ca^2+^ dynamics are subject to tight spatiotemporal regulation by opening of Ca^2+^ channels, and buffering and removal of Ca^2+^ ions^1^. Two major sources of Ca^2+^ in the cytosol are those entering from the outside milieu, and intracellular stores such as the endoplasmic reticulum, mitochondria, and nucleus. Extracellular Ca^2+^ signalling has been extensively studied in excitable cells with a large negative membrane potential, including neurons, glia, and muscle^2–5^. In contrast, Ca^2+^ signalling in non-excitable cells is mainly associated with release of Ca^2+^ from intracellular stores, while the role of extracellular Ca^2+^ signalling in non-excitable cells remains largely obscure.

Depolarisation-induced influx of extracellular Ca^2+^ in excitable cells occurs through two key types of Ca^2+^ channel, namely voltage-gated Ca^2+^ channels (VGCCs) and NMDA-type glutamate receptors (NMDARs)^6,7^. In neurons, VGCC and NMDAR signalling carries important functions including controlling membrane trafficking and gene expression. Signalling via NMDARs in particular underscores the mechanisms of synaptic plasticity, memory and learning. Dysregulated NMDAR function is implicated in a large variety of CNS disorders, including neurodegeneration, stroke, schizophrenia, and addiction^8–10^. Because of this, NMDARs have been extensively investigated in the central nervous system.

NMDAR channel opening is generally believed to require simultaneous binding of its agonist glutamate and depolarisation of the membrane, which represents is a tightly orchestrated instance in neurons, where ambient glutamate levels are low and the plasma membrane is highly polarised. However, spontaneous agonist-independent opening of NMDARs has also been reported^11^. Conversely, high levels of extracellular glutamate (around 50 μM) and weakly polarised cell membranes^12^ in the peripheral tissues suggest that NMDARs outside of CNS may be tonically active, with potentially important functional consequences. The notion of functional relevance of peripheral NMDARs is further supported by their expression in peripheral tissues and upregulation in several cancers^13–15^ and the anti-tumour effect of NMDAR antagonists^15–17^. Taken together, these considerations imply that NMDARs may indeed play a role in cellular function – and dysfunction – outside the  CNS. This role, however, remains unexplored.

This study sought to determine the role of extracellular Ca^2+^ signalling on membrane trafficking regulation in peripheral cell types, using well-characterised pharmacological tools, membrane trafficking assays and confocal microscopy. Its results show that NMDARs – but not VGCCs – couple extracellular Ca^2+^ influx with membrane trafficking and organisation of early endosomes (EE). Remarkably, NMDARs differentially regulate membrane trafficking and endosomal structure in a cancer cell line. These findings indicate that NMDAR signalling has a fundamental role in cells beyond the CNS, and implicate membrane trafficking as a potential cell biological mechanism linking glutamate signalling and cancer.

## Results

### Ca^2+^ influx through NMDA receptors regulates endosomal structure

**Figure 1:**
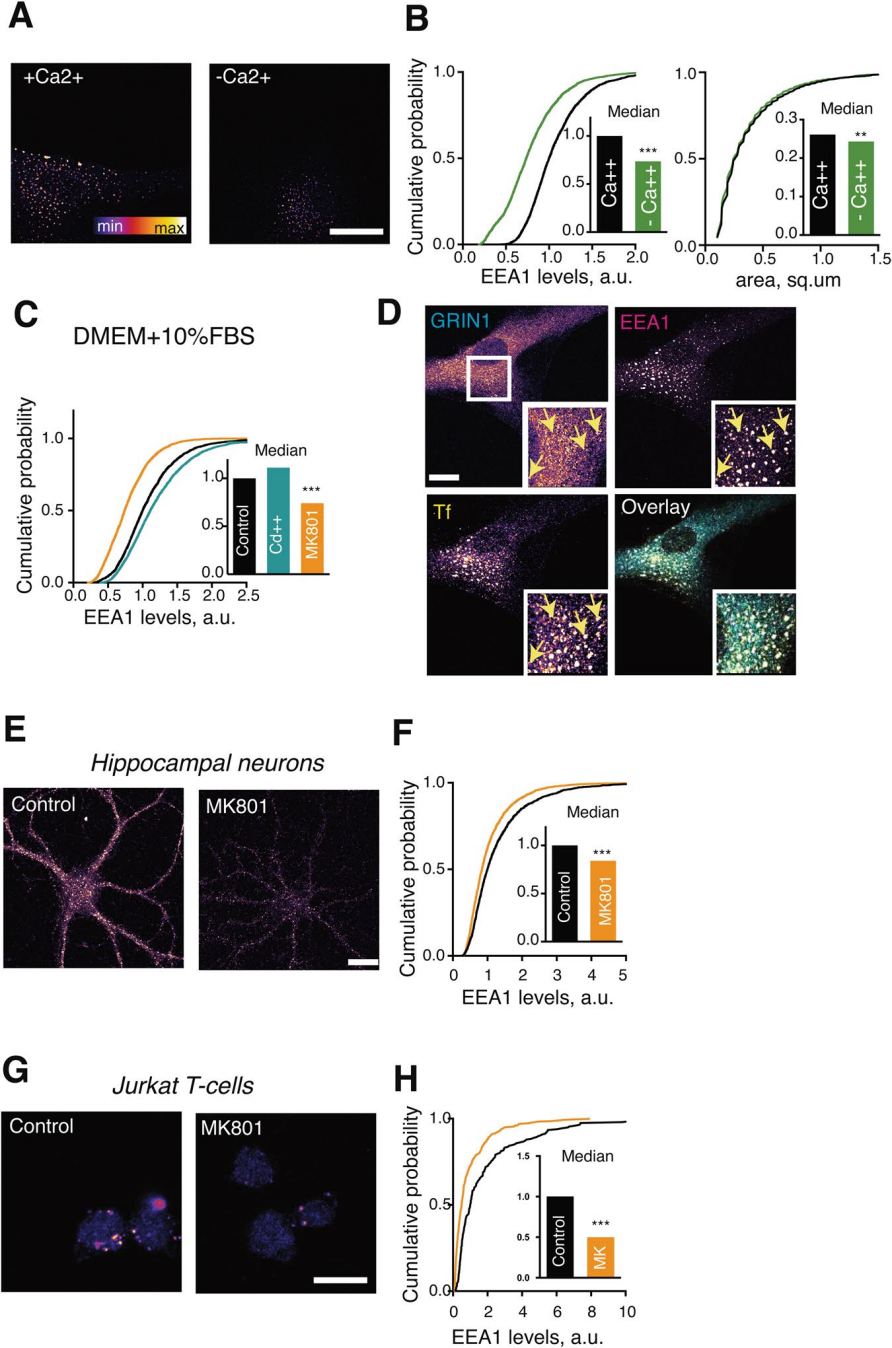
Ca^2+^ influx through NMDARs rapidly regulates EEs. (**A**) Representative image of primary human fibroblasts incubated for 10 min in PBS or PBS supplemented with 1.8 mM CaCl_2_. (**B**) Left, quantification of EEA1 immunostaining levels in cells incubated in  PBS with or without added CaCl2. Right, quantification of the EEA1 area in cells incubated in  PBS with or without added CaCl_2_ . N = 50–250 EEs/image, 20 images/condition, 4 independent experiments. (**C**) Quantification of the EEA1 immunostaining levels in cells following incubation for 10 min in DMEM + 10% FBS in presence of indicated drugs. N = 50–250 EEs/image, 25 images/condition, 5 independent experiments. (**D**) Immunostaining for  endogenous GRIN1 in fibroblasts. Live cells were incubated with fluorescent Transferrin (Tf) for 1 h at 37 °C, fixed and immunostained for GRIN1 and EEA1. Arrows denote GRIN1 signal colocalising with EEA1- and Tf-positive puncta. (**E**) Dissociated rat hippocampal neurons (21 days *in vitro*) were treated with MK801 for 30 min at 37 °C. (**F**) Quantification of the EEA1 immunostaining levels in neurons treated with MK801. N = 20–100 EEs/image, 15 images/condition, 3 independent experiments. ***P < 0.0001, **P < 0.01, Mann-Whitney U test. Scale bar, 20 μm. (**G**) Jurkat T cells were treated with MK801 for 30 min at 37 °C. (**H**) Quantification of the EEA1 immunostaining levels in Jurkat T cells treated with MK801. N = 20–100 EEs/image, 3–5 images/condition, 2 independent experiments. ***P < 0.0001, Mann-Whitney U test. Scale bar, 20 μm.

**Figure 2:**
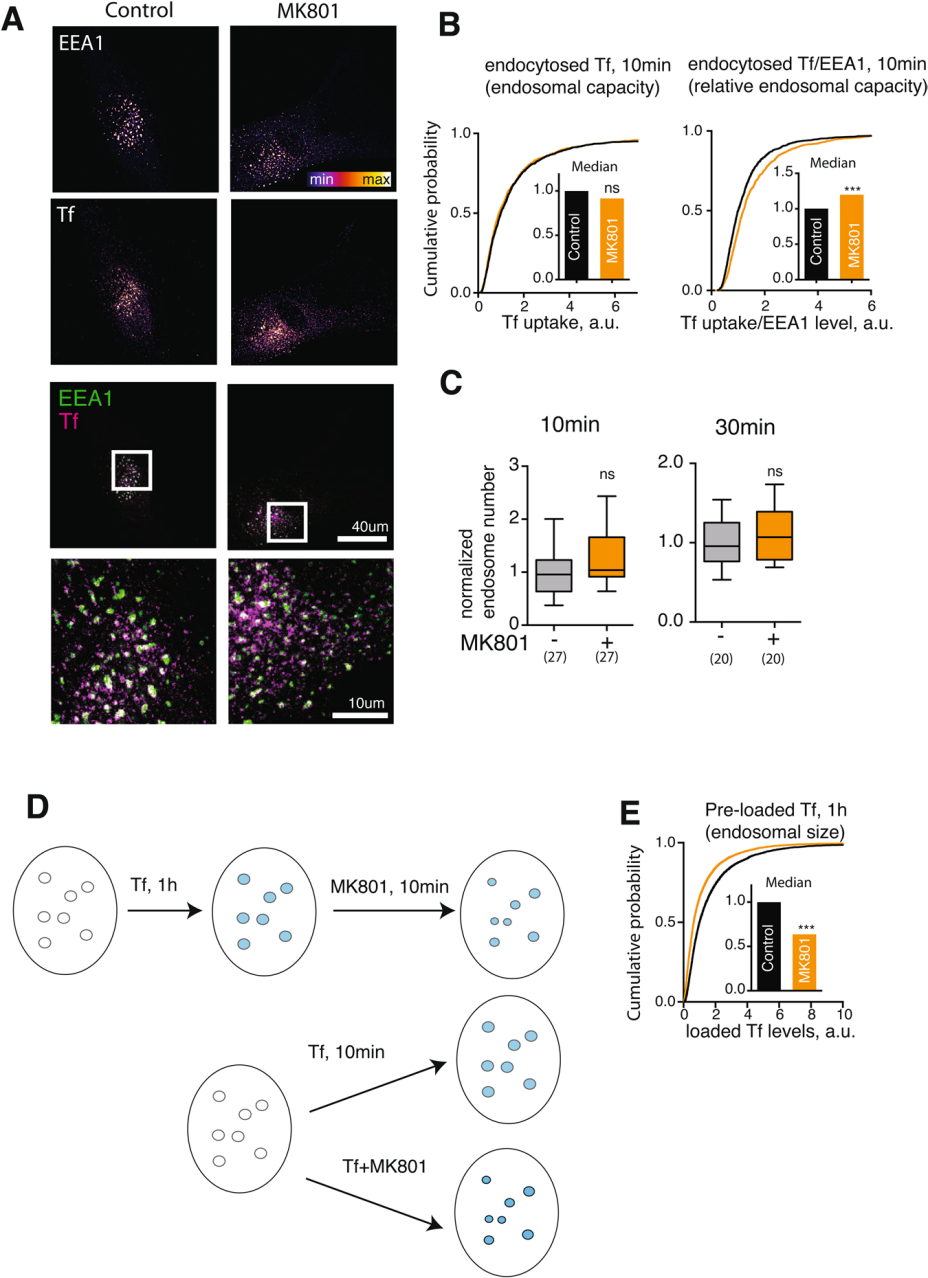
Regulation of trafficking through early endosomes by NMDAR signalling. (**A**) Representative image of fibroblasts incubated with fluorescent Tf in presence of MK801 for 10 min. Note the colocalisation of the internalised Tf signal with EEA1-positive puncta. (**B**) Quantification of the Tf level in EEs following MK801 treatment. Left, quantification of absolute Tf levels. Right, quantification of the Tf levels divided by the EEA1 levels. ns – not significant, ***P < 0.0001, Mann-Whitney U test. N = 50–250 EEs/image, 15 images /condition, 3 independent experiments. (**C**) Normalised endosome number per cell following MK801 treatment for 10 and 30 min. ns = not significant, Student’s t test. N = 15 cells/condition, 3 independent experiments. (**D**) Schematic for the two Tf uptake experiment. Top, pre-loading with Tf for 1 hour prior to the MK801 treatment allows to quantify the effect of NMDAR blockade on the EEs already containing Tf, *i.e*. functionally active endosomes. Bottom, applying Tf simultaneously with MK801 treatment allows to quantify the effect of NMDAR blockade on Tf endocytic rate. (**E**) EE-specific levels of pre-loaded Tf following MK801 treatment. ***P < 0.0001, Mann-Whitney U test. N = 10 images/condition, 2 independent experiments.

**Figure 3:**
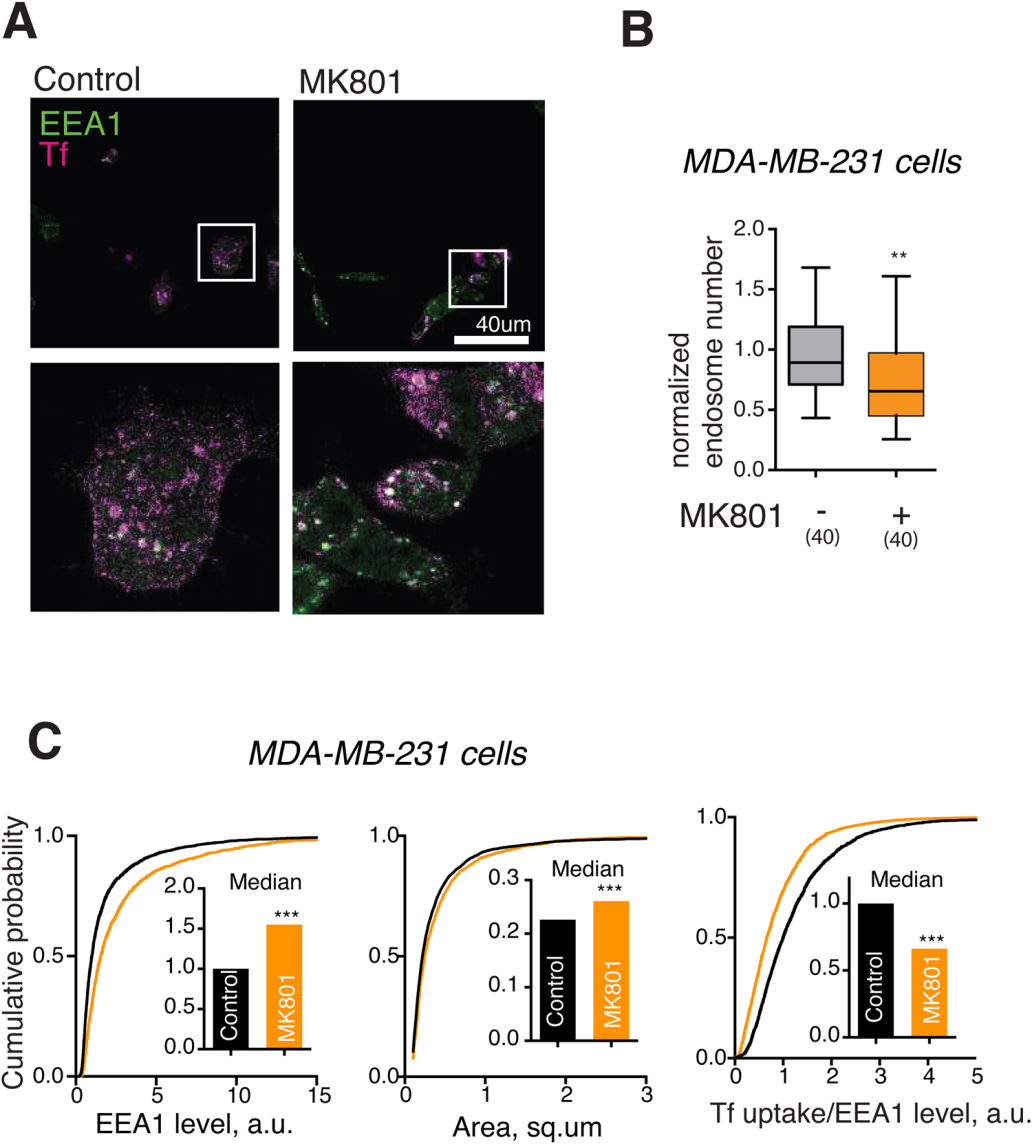
Aberrant regulation of EE structure by NMDARs in a cancer cell line. (**A**) Representative image of MDA-MB-231 cells incubated with fluorescent Tf in presence of MK801 for 30 min. N = 4 independent experiments. (**B)** Normalised endosome number per a MDA-MB-231 cell following MK801 treatment for 30 min. **P < 0.01, Mann-Whitney U test, 40 cells/condition, 4 independent experiments. (**C)** Effects of MK801 treatment on EE area, EE-specific EEA1 levels, EE-specific Tf/EEA1 ratio. P < 0.0001, Mann-Whitney U test. N = 50–200 EEs/image, 25 images/condition, 5 independent experiments.

EE structure was visualised and quantified using immunostaining for the membrane-binding protein early endosome antigen 1 (EEA1), which is exclusively enriched in EEs. As expected, EEA1 immunostaining invariably presented a strongly punctate pattern in all the cell types employed in this study, consistent with its designation as a canonical and well-established marker for functional EE (Figs. 1–3**)**. Interestingly, EEA1 puncta in primary human fibroblasts incubated in phosphate saline buffer (PBS) with or without added 1.8 mM CaCl_2_ exhibited different morphologies, namely omission of Ca^2+^ from the buffer resulted in a decrease in EEs as manifested both by a decrease in the median EE-specific levels of EEA1 staining and a decrease in the median area of the EEs; the effect was noticeable within 10 min of incubation (Fig. 1A,B). This suggested that extracellular Ca^2+^ levels were somehow linked to the morphology of the endosomal system; therefore, it was hypothesised that this regulation is controlled by the influx of extracellular Ca^2+^.

Extracellular Ca^2+^ can regulate intracellular processes either through direct influx into the cell, or indirectly through Ca^2+^-binding cell surface molecules such as cadherin^18^. To test whether Ca^2+^ channels were involved in regulation of EEs, VGCCs and NMDARs were pharmacologically blocked in fibroblasts maintained in Ca^2+^-containing standard culture medium complemented with serum. To this end, a non-selective VGCC inhibitor CdCl_2,_ and an open-pore NMDAR blocker MK801 were used in concentrations previously shown to block their function in neurons (100 μM and 25 μM respectively)^19,20^. Only the effect of MK801 treatment mimicked that of Ca^2+^ free medium (Fig. 1C). This observation suggested that (a) endosomes were likely to be regulated by Ca^2+^ influx rather than by cell adhesion pathways, and (b) NMDAR rather than VGCC signalling was required to maintain EEs.

Do fibroblasts express functional NMDARs? NMDAR mRNA has been detected in fibroblasts^21^, and early studies show a sufficiently depolarized membrane potential of −6 to −16 mV^22^. To complement the published data, expression of NMDARs was confirmed using immunocytochemistry for a major NMDAR subunit GRIN1. Staining for GRIN1 was present throughout the cell body, including the plasma membrane and the EEA1 endosomes labelled for EEA1 (Fig. 1D**)**. Given that the concentration of the main endogenous NMDAR agonist glutamate under standard culture conditions (DMEM/F-12 + 10% FBS) is in the region of 87 μM (Büntemeyer *et al*., 1991) *i.e*. an order of magnitude above the EC50 for NMDAR activation, NMDARs on the plasma membrane of the cultured fibroblasts are likely to be constitutively active. Consistent with this notion, addition of the canonical NMDAR agonist N-methyl-D-aspartate (NMDA) (50 μM) had no further effect on EEs (data not shown), suggesting that NMDARs are likely to be already active.

Considering the key specialised roles of NMDARs in neuronal function^23^, the effect of NMDAR blockade on EEs was also studied in cultured rat hippocampal neurons. 30 min blockade of NMDARs by MK801 modestly reduced EEs, although the effect was less pronounced than in fibroblasts (Fig. 1G,H). The effect of MK801 on EEs was also noted in Jurkat T cells, where NMDAR signalling has been previously implicated in cell growth and adhesion^24^; expression of GRIN1 was further confirmed by immunostaining (data not shown). Therefore, NMDAR signalling may regulate endosomal structure in neurons as well as in peripheral tissues.

### NMDAR signalling in fibroblasts regulates endosomal morphology but not endocytic uptake

The effects of NMDAR blockade on EEs were investigated in more detail. Given that MK801 may block other receptors besides NMDARs *e.g*. nicotinic acetylcholine receptors^25^, the effect of an unrelated NMDAR antagonist (2 R)-amino-5-phosphonovaleric acid (APV) was assessed. Similarly to MK801, the median intensity of the EEA1 puncta rapidly decreased following a 10-minute incubation with 100 μM APV, as was the median EE area (Fig. S1A,B). A similar result was obtained when another, also unrelated, NMDAR antagonist memantine (50 μM) was used (Fig. S1C,D). Thus, the observed effect on EE is likely to be due to specific inhibition of NMDAR signalling.

To assess EE function following NMDAR blockade, I investigated the effect of NMDAR blockade on trafficking of a canonical endocytic cargo protein transferrin (Tf) to EE. Following endocytosis from the cell membrane, Tf is rapidly enriched in EEs^26^. Accordingly, following 10 min of incubation, Tf  colocalised with the EEA1-positive puncta, further confirming that they corresponded to functional EEs (Fig. 2A). The absolute levels of Tf internalised into EEs were unaffected by MK801, indicating that NMDAR did not regulate the endocytic rate; indeed, levels of internalised Tf was significantly enriched in EEs relative to the EEA1 content following MK801 treatment (Fig. 2B). The number of endosomes per cell was not significantly altered (Fig. 2C).

To confirm the effect of NMDAR blockade on EE with an alternative marker and to establish whether NMDAR blockade affected functionally active EEs, I pre-loaded fibroblasts with fluorescently labelled Tf for 1 hour before the pharmacological treatment (Fig. 2D). The effect of NMDAR blockade was then assessed by quantifying the Tf signal within the EEA1-positive puncta following the treatment. Tf levels were decreased by the MK801 treatment to an extent comparable to that of EEA1 (Fig. 2E), further confirming that NMDAR blockade affected EEs .

### Differential regulation of endosomal morphology in cancer-associated cell lines

Functional NMDARs have been reported in multiple cancer cell lines^13,14,17,27^, and aberrant glutamate signalling through NMDARs has been implicated as a factor in some forms of cancer^16^. Blockade of NMDAR slows down growth in models of breast, pancreatic and prostate cancer^16,28^. To investigate the link between NMDAR signalling and EE structure in cancer, the effect of NMDAR blockade on EEs was investigated in two cancer cell lines – prostate cancer-associated fibroblasts (CAFs) and a breast cancer cell line, MDA-MB-231.

Application of MK801 in CAFs resulted in a similar effect to that observed in wild type fibroblasts, *i.e*. EEA1 content per endosome was decreased while functionality was retained. Although the median area of EE was not decreased, the distribution of areas was significantly different between the control and MK801-treated cultures, indicating that NMDAR blockade still affected the structure of EE (Fig. S2). In contrast, MK801 treatment of MDA-MB-231 cells had an opposite effect, significantly increasing both the area of EE and the endosomal levels of EEA1, while the number of endosomes per cell and the internalised Tf levels relative to the endosomal EEA1 were decreased (Fig. 3A,B). This indicated that NMDAR signalling affected EE organisation in MDA-MB-231 cells in a manner distinct from that in fibroblasts.

## Discussion

Unlike the role of glutamate receptor signalling in the brain, which has been extensively investigated, little is known about the role of glutamate receptor signalling outside of the CNS. This short study reports a new cell biological function for glutamate receptor signalling outside of the CNS, showing that NMDAR signalling is coupled to organisation of the endosomal system in several types of mammalian cells. Moreover, coupling of NMDAR activity  with endosomal structural dynamics provides conceptual evidence that extracellular Ca^2+^ signalling may control structural aspects of intracellular organisation. The rapid timescale (10 min) of the reported effect is consistent with an effect of Ca^2+^ influx on EE structure that relies on signalling mechanisms rather than slower regulatory modalities of gene expression.

The small membrane potential of electrically passive cell types may favour opening of VGCCs as well as NMDARs; indeed, early studies hint at the presence of functional VGCCs in fibroblasts^29,30^. However, blockade of VGCCs did not diminish the EE structure, indicating that tonic Ca^2+^ signalling through VGCCs is either negligibly small or functionally uncoupled from the NMDAR signalling, as is the case in neurons^31^. The relative contribution of VGCCs to Ca^2+^ dynamics in non-excitable cells and the functional ramifications of VGCC signalling outside of the CNS remain to be explored.

Comparatively high concentrations of glutamate and glycine along with low negative polarisation of the plasma membrane in peripheral tissues are in contrast to the CNS, where membranes of both neurons and glia are highly polarised and the concentration of free glutamate is kept low. Nevertheless, our data shows that blockade of NMDAR affects endosomal organisation in neurons as well as in non-neuronal cell types (Fig. 1G,H), suggesting that regulation of endosomal structure by glutamate receptor signalling may be a general feature of mammalian cells. In addition to NMDAR activation through agonist and co-agonist binding, NMDAR signalling may also involve agonist-independent spontaneous opening^11,32^. Further investigation will focus on detailed aspects of NMDAR pharmacology in peripheral tissues, such as the relative contribution of agonist-induced and spontaneous opening, as well as the role of co-agonist binding.

EE is an important regulatory nexus for functional cross-talk between signalling pathways^33^. Therefore, control of EE structure dynamics may serve as a structural mechanism allowing NMDAR activation to exert control over its key downstream effectors such as the ERK/MAPK pathway, which is known to be regulated by EE sorting^34^. Further work will be required to identify the mechanistic details of the coupling mechanisms between NMDAR-dependent Ca^2+^ influx and EE dynamics.

Aberrant NMDAR signalling is implicated in several major types of cancer^13–17,28^. The distinct effect of NMDAR blockade on EEs in a breast cancer cell line (Fig. 3) indicates that aberrant glutamate signalling may contribute to carcinogenesis through dysregulation of endosomal dynamics, consistent with the well-established involvement of endosomal membrane trafficking in cancer^35^. It would be premature to speculate on the mechanistic basis for this dysregulation, as MDA-MB-231 is a highly divergent cancer cell line, with 2948 genes showing altered copy number and 946 genes showing altered expression^36^; however, some of these can be linked to NMDARs and Ca^2+^ signalling, e.g. an NMDAR-anchoring protein Map1A and a number of calmodulin-like proteins. Correcting the defects of NMDAR-dependent membrane trafficking may therefore offer a potential avenue for therapeutic development in cancer.

In conclusion, this study shows an unexpected cell biological function for NMDAR signalling in peripheral tissues. Taken in the broader context of cell biology, these results raise multiple questions regarding the fundamental importance of NMDAR and glutamate receptor signalling in general. What is the mechanism transducing NMDAR-dependent Ca^2+^ influx into EE dynamics? Which subunits of NMDAR form functional channels in peripheral cells? Does NMDAR signalling regulate other endomembrane compartments? How does extracellular Ca^2+^ influx interact with other forms of intracellular Ca^2+^ signalling in non-neuronal cell types? How evolutionarily conserved is the coupling between glutamate signalling and endosomal dynamics, and do other glutamate receptors regulate the cell biology of non-excitable cells? Finally, what is the functional relevance of NMDAR-dependent EE dynamics in peripheral cells, and how does it go awry in cancer? Finding the answers to all these questions will warrant extensive further investigation.

## Materials and Methods

### Reagents

Fetal calf serum, Dulbecco’s Minimal Essential Medium (DMEM) low-glucose medium and Roswell Park Memorial Institute (RPMI) medium were from Gibco (UK). Anti-EEA1 and anti-GRIN1 antibody were from Abcam (UK). Secondary antibodies were from Jackson Immunoresearch (USA). AlexaFluor555-conjugated Transferrin was from Thermo Fisher (UK). APV, MK801, memantine were from Tocris (UK). CdCl_2_, CaCl_2_ and FITC-cholera toxin B subunit were from Sigma-Aldrich (UK). Fluoromount-G mounting medium was from Southern Biotech (USA).

### Cell culture

Human fibroblasts and MD-MB-231 cells were grown in DMEM low-glucose medium with 10% fetal calf serum. Jurkat cells were grown in RPMI medium with 10% fetal calf serum. Dissociated hippocampal neuronal cultures were prepared from E18 rat embryos and grown according to the Banker protocol in Neurobasal medium with added GlutaMax and B-27 supplement. All experiments involving neurons were carried out at 16–21 days *in vitro*. All cell lines apart from Jurkat were plated onto 13 mm round glass coverslips (thickness 1.5) placed in 35 mm Petri dishes (4 coverslips/dish). Jurkat cells were grown in suspension and allowed to attach to the coverslips for 30 min immediately before the experiment. To reduce variability, each experiment was carried out using the coverslips cultured within the same Petri dish.

### Ethics Statement

Hippocampal neuronal cultures of rat neurons were prepared using procedures approved by the King’s College London Ethical Review Process Committee. Human fibroblast cultures were derived from the samples obtained under clinical approvals No. 09/H0807/089 and 06/Q0406/33, as described elsewhere^37^. All experiments involving primary human cell lines were carried out in accordance with the Human Tissue Act (2004). All methods were performed in accordance with the relevant guidelines and regulations.

### Immunostaining and confocal microscopy

After treatment, coverslips were fixed with 4% paraformaldehyde in PBS for 15  min at room temperature (RT) and permeabilised in 0.3% Triton-X100 in PBS supplemented with 5% horse serum for 10 min. Subsequent incubations were carried out in the permeabilisation buffer. Coverslips were incubated with appropriate primary antibodies for 60 min at RT, washed 4 times in PBS and incubated with AlexaFluor488, AlexaFluor568- and AlexaFluor647-conjugated secondary antibodies as appropriate at a concentration of 0.3 μg/mL each for 60 min at RT. Coverslips were then mounted in Fluoromount-G mounting medium and imaged on a Zeiss LSM710 microscope equipped with a standard set of lasers through a Plan-Apochromat 63×/1.4 Oil objective. The imaging system was controlled by ZEN software. To minimise observational bias in quantification, cells were visualised using the general membrane staining by FITC-Choleratoxin B subunit, blind with respect to the other channel(s). Regions of interest sized 1024×1024 pixels (65.8 nm/pixel) were imaged at speed 7 with the averaging setting 2. Pinhole size was set to 1 Airy unit. Excitation laser wavelengths were 488 nm, 543 nm and 633 nm. Bandpass filters were set at 500–550 nm (AlexaFluor488), 560–615 nm (Cy3, AlexaFluor568) and 650–750 nm (AlexaFluor647). Image acquisition was carried out at the 12-bit rate. Settings were optimised to ensure appropriate dynamic range, low background and sufficient signal/noise ratio. For Tf uptake assay, cells were incubated with 1/100 AlexaFluor555-conjugated Tf in culture medium for 10 min or 1 hour at 37 °C, then fixed and processed for immunostaining and confocal microscopy as detailed above.

### Image analysis

To identify individual EEs, images were binarised in ImageJ using the “Moments” setting, and particles were counted automatically using the “Analyze Particles” command across the whole image. Binarised data from EEA1 immunostaining channel was used for determination of EEs. Signal intensities were quantified for each EE using the Region Of Interest (ROI) Manager function of ImageJ. To avoid rare overlap of multiple endosomes, only ROIs with areas ranging from 0.1 to 2 μm^2^ were included in further analysis. All values of circularity were included in analysis. Since background fluorescence intensity was typically less than 1% of the median ROI fluorescence, background subtraction did not significantly affect the measurements and was not performed.

For quantification of absolute Tf levels in EEs, ROIs were defined as the areas with EEA1 punctate staining as described above. For quantification of relative Tf levels in EEs, these values were then divided by the corresponding values for EEA1 intensity in the corresponding areas. In each experiment, all intensity values were normalised to the median value in the control sample. Values for areas were not normalised.

### Statistical analysis

All the experiments were performed in 2–5 independent biological replicates, with five fields of view per condition per replicate selected for analysis. All of the EEs automatically detected within these fields of view were included in the analysis. Statistical analysis was carried out using the Prism 6.0c software package (GraphPad Software). Data distributions were assessed for normality using d’Agostino and Pearson omnibus normality test. Mann-Whitney rank test was used for assessing statistical significance, and the entire dataset was presented as cumulative probability plots and median levels.

## Supplementary information

Supplementary information.
