# Supplementary material for: Tonic NMDA receptor signalling shapes endosomal organisation in mammalian cells
Source: Sci Rep. 2020 Jun 9;10:9315. doi: 10.1038/s41598-020-66071-0 (PMC7283358; doi:10.1038/s41598-020-66071-0)
Supplement: Supplementary file 1 — Supplementary information. [file 41598_2020_66071_MOESM1_ESM.docx]

# Tonic NMDA receptor signalling shapes endosomal organization in mammalian cells (Supplementary Information)

Short title: NMDA receptor function in non-excitable cell types

Oleg O. Glebov^1,2^

^1^ Institute of Neuroregeneration and Neurorehabilitation, Qingdao University, Qingdao 266071, Shandong, China.

^2^ Department of Old Age Psychiatry, The Institute of Psychiatry, Psychology & Neuroscience, King’s College London, De Crespigny Park, Denmark Hill, London SE5 8AF, UK.

E-mail: oleg.glebov@kcl.ac.uk

**Figure S1. Two other NMDAR antagonists besides MK801 also decrease EEs in fibroblasts. A,** Primary human fibroblassts were treated with 100 μM APV for 10 min. **B**, Effects of APV treatment on EE-specific EEA1 levels and EE area. ***P<0.0001, Mann-Whitney U test. N=40-250 EEs/image, 15 images/condition, 3 independent experiments. **C**, Primary human fibroblasts were treated with 50 μM Memantine for 10 min. **D**, Effects of Memantine treatment on EE-specific EEA1 levels and EE area. ***P<0.0001, Mann-Whitney U test. N=40-150 EEs/image, 10 images/condition, 2 independent experiments. Scale bar, 40 μm.

**Figure S2. Regulation of EE structure by NMDARs in a cancer-associated fibroblast cell line.** CAFs were incubated with MK801 for 30 min. Effects of MK801 treatment on EE-specific EEA1 level and Tf/EEA1 ratio are shown. P<0.0001, Mann-Whitney U test. N=40-200 EEs/image, 20 images/condition, 4 independent experiments.
